# Case Report: Early introduction of anti-GD2 immunotherapy during induction chemotherapy in high-risk neuroblastoma: two pediatric cases

**DOI:** 10.3389/fonc.2026.1863412

**Published:** 2026-08-04

**Authors:** Tianjiao Hu, Jian Li, Lulu He, Xiaojun Zhang, Jin Xu, Tao Li, Li Zhou

**Affiliations:** 1 Department of Pharmacy, Children’s Hospital of Nanjing Medical University, Nanjing, China; 2 Department of Oncology, Children’s Hospital of Nanjing Medical University, Nanjing, China; 3 Department of Hematology, Children’s Hospital of Nanjing Medical University, Nanjing, China; 4 Department of Radiology, Children’s Hospital of Nanjing Medical University, Nanjing, China

**Keywords:** anti - GD2, case report, induction chemotherapy, naxitamab, neuroblastoma, pediatric

## Abstract

Anti-Ganglioside (GD)2 monoclonal antibodies are widely used as maintenance therapy for high-risk neuroblastoma (HR-NB). However, the potential benefit of introducing immunotherapy during induction chemotherapy remains unclear. We report two pediatric patients with newly diagnosed HR-NB who received naxitamab in combination with chemotherapy starting from the second cycle of induction. Clinical characteristics, treatment regimens, and outcomes were retrospectively reviewed. Both patients achieved complete remission (CR) per International Neuroblastoma Response Criteria at the end of induction. Treatment-related adverse events, including hypotension, pain, and rash, capillary leak syndrome, and chemotherapy-related myelosuppression, were transient and manageable with supportive care and infusion adjustments. No treatment discontinuation due to toxicity occurred. While these preliminary findings demonstrate the feasibility and tolerability of early naxitamab introduction, the limited sample size precludes definitive conclusions regarding efficacy. This report provides exploratory data to support future prospective studies on optimizing the timing of anti-GD2 immunotherapy in HR-NB.

## Introduction

Neuroblastoma (NB) is the most common extracranial solid tumor in children and accounts for approximately 8–10% of pediatric malignancies (1, 2). The majority of cases (80%–90%) are diagnosed before five years of age (3). High-risk neuroblastoma (HR-NB) frequently presents with metastatic disease and remains associated with poor outcomes despite intensive multimodal therapy (4). The introduction of GD2-targeting monoclonal antibodies as post-consolidation maintenance therapy has markedly improved outcomes, with overall survival now exceeding 50% (5, 6).

However, the optimal timing for anti-GD2 antibody administration remains uncertain. Preclinical and clinical evidence suggest that both tumor burden and host immune competence critically influence therapeutic efficacy. The induction phase, during which tumor burden is substantially reduced and immune function has not yet been compromised by high-dose chemotherapy (HDC) and autologous stem cell transplantation (ASCT), may represent a particularly favorable window for anti-GD2 immunotherapy. Here, we report two cases of HR-NB treated with early introduction of naxitamab during induction chemotherapy, highlighting the feasibility, safety, and clinical outcomes of this approach.

## Case presentation

We report two newly diagnosed high-risk neuroblastoma patients who received naxitamab combined with chemotherapy starting from the second cycle of standard induction. Both patients were treated with the 2021 pediatric neuroblastoma induction protocol comprising six 21-day cycles. Cycles 1 and 2 consisted of cyclophosphamide (400 mg/m²/day, days 1–5) plus topotecan (1.2 mg/m²/day, days 1–5). Cycles 3 and 5 consisted of cis-platinum (50 mg/m²/day, days 1–4) plus etoposide (200 mg/m²/day, days 1–3). Cycles 4 and 6 consisted of cyclophosphamide (2100 mg/m²/day, days 1–2), mesna (420mg/m^2^, 4 hours/dose, three times days 1–2), doxorubicin (25 mg/m²/day, days 1–3), plus vincristine (0.67 mg/m²/day, days 1–3). Naxitamab (2.25 mg/kg/day) was administered on days 2, 4, 8, and 10 of Cycles 2 through 6. Both patients completed the planned induction courses and achieved complete remission (CR) by the International Neuroblastoma Response Criteria (INRC) at end of treatment.

### Patient 1

A 4-year-old girl presented with fever and was found to have a retroperitoneal mass with multiple metastatic lesions. She had a history of purulent meningitis at one month of age, from which she had completely recovered.

Initial laboratory evaluation revealed a white blood cell count of 5.65 ×10^9^/L, hemoglobin of 106 g/L, platelet count of 455 ×10^9^/L, and neutrophils count of 3.66 ×10^9^/L. Lactate dehydrogenase was elevated at 531 U/L. Serum neuron-specific enolase (NSE) was markedly increased to 163.0 ng/mL (reference range: 1–16.3 ng/mL). Urinary vanillylmandelic acid (VMA) and homovanillic acid (HVA) data were not available for this retrospective review.

**Figure 1:**
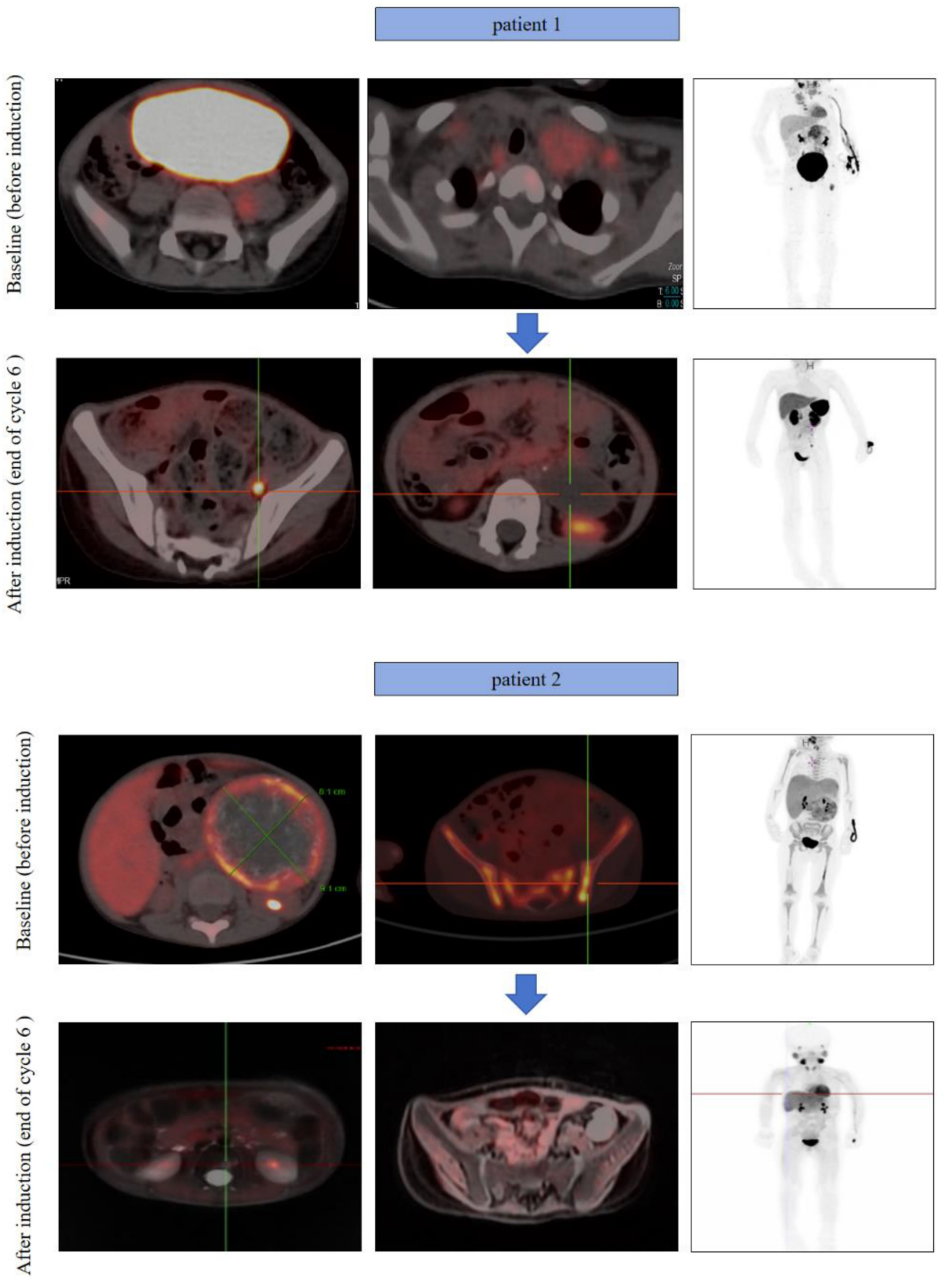
Radiological response before and after induction therapy in two patients with high-risk neuroblastoma. Baseline imaging demonstrated extensive primary tumors and metastatic lesions, while post-induction imaging showed marked regression. Different imaging tracers were used due to clinical practice considerations. Patient 1 underwent baseline imaging with 18F-mFBG and follow-up imaging with DOTA-PET/CT, whereas Patient 2 underwent baseline imaging with 18F-FDG PET/CT and follow-up imaging with 18F-mFBG. Despite these differences, both patients showed consistent and significant reduction in tumor burden.

Contrast-enhanced imaging at diagnosis revealed a large retroperitoneal mass with metastatic involvement of lymph nodes and bones. Baseline 18F-mFBG PET/CT demonstrated extensive tracer uptake in the primary lesion and metastases (**Figure 1**). ^123^I-MIBG scintigraphy showed widespread uptake with a Curie score of 8 (reference standard for bone metastasis burden). Bone marrow biopsy confirmed neuroblastoma infiltration. Molecular profiling identified non-amplified MYCN, 11q deletion, ATRX loss, and concurrent CDK4 and MDM2 amplification. Based on the International Neuroblastoma Risk Group (INRG) classification, the patient was diagnosed with stage M high-risk neuroblastoma.

The patient received the induction regimen detailed above. Premedication with gabapentin was initiated 5 days prior to each naxitamab cycle, and granulocyte-macrophage colony-stimulating factor (GM-CSF) was administered on days 6–10 of each cycle. During the first naxitamab course (Cycle 2 of induction), the patient developed infusion-related adverse events, including grade 3 generalized pain, as well as grade 2 hypotension, pruritic rash, and capillary leak syndrome, all of which resolved with supportive care and temporary infusion rate adjustments. These events recurred with similar severity during the subsequent 4 courses (Cycles 3–6) and were managed with predefined supportive measures. In addition, chemotherapy-related myelosuppression, manifesting as leukopenia and anemia, occurred during the post-chemotherapy recovery phase and was managed with rhGM-CSF and prophylactic antibiotics, with blood counts recovering prior to the next cycle. Detailed toxicity data are provided in **Table 1**.

**Table 1:** Treatment-related adverse events graded per CTCAE v5.0.

Patient	Adverse event	Grade	Intervention	Outcome
Patient 1	Generalized pain	3	IV fluids, analgesics, rate adjustment	Resolved within 12 h
	Hypotension	2	IV fluid bolus, rate adjustment	Resolved within 2 h
	Pruritic rash	2	Antihistamines	Resolved within 2 h
	Capillary leak syndrome	2	IV albumin, supportive care	Resolved within 48 h
	Febrile neutropenia/myelosuppression	3	rhGM-CSF, prophylactic antibiotics	Resolved prior to next cycle
Patient 2	Abdominal pain	2	IV fluids, analgesics	Resolved within 6 h
	Tachycardia	2	Rate adjustment, supportive care	Resolved within 2 h
	Irritability	2	Supportive care	Resolved within 2 h
	Hypotension	2	IV fluid bolus, rate adjustment	Resolved within 2 h
	Myelosuppression	3	rhGM-CSF, supportive transfusions	Resolved prior to next cycle

CTCAE, Common Terminology Criteria for Adverse Events; IV, intravenous; rhGM-CSF, recombinant human granulocyte-macrophage colony-stimulating factor.

**Figure 2:**
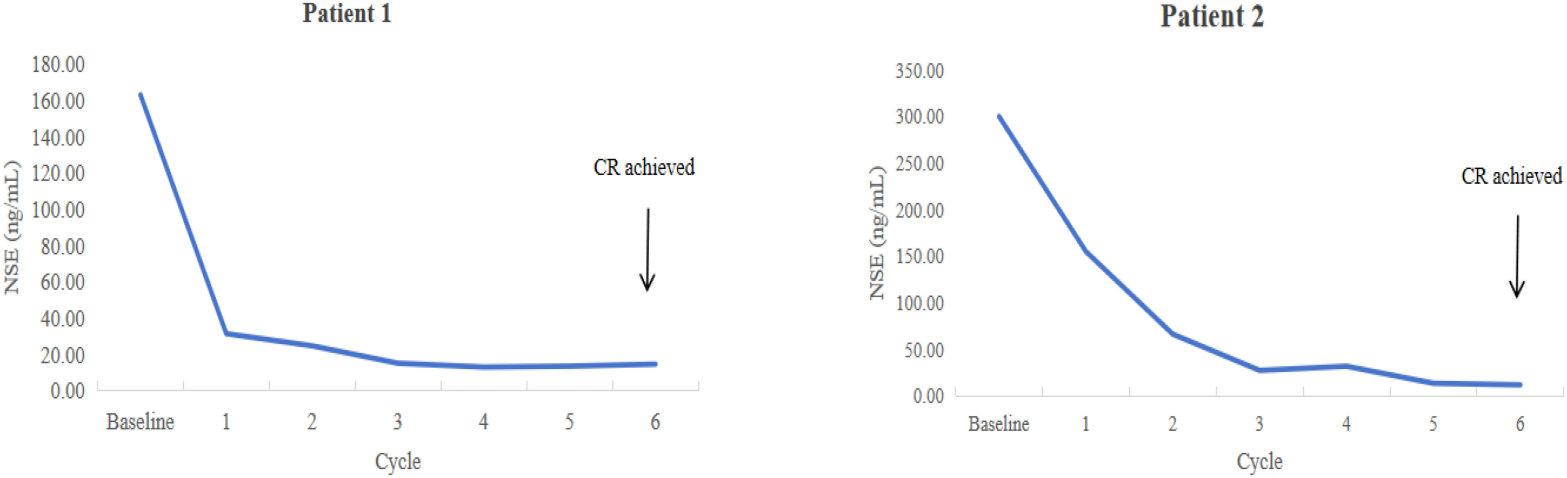
Changes in serum neuron-specific enolase (NSE) levels during induction therapy in two patients. Both patients demonstrated a rapid decline in NSE levels during treatment, reaching normal levels by the end of induction, consistent with imaging findings and clinical response.

Following six cycles of induction therapy, her NSE normalized to 14.6 ng/mL (**Figure 2**). Post-induction ¹²³I-MIBG scintigraphy demonstrated a Curie score decrease from 8 to 0, and DOTA-PET/CT showed marked regression of the primary tumor and nodal lesions. Multiparameter flow cytometry of bone marrow (sensitivity: 0.01%) was negative for minimal residual disease. Based on concordant findings across imaging, MIBG, bone marrow, and biochemical markers, the patient achieved complete remission per International Neuroblastoma Response Criteria (INRC) at the end of induction.

The induction phase concluded on December 25, 2025. She subsequently underwent involved-field radiotherapy to the primary tumor bed in February 2026 (total dose 21.6 Gy), followed by single autologous hematopoietic stem cell transplantation in May 2026 after conditioning with busulfan and melphalan. She is currently continuing consolidation therapy with naxitamab maintenance. As of the latest follow-up (6 months post-induction), there has been no evidence of relapse.

### Patient 2

A 3-year-old girl presented with a 2-day history of fever and abdominal distension. Initial laboratory evaluation revealed a markedly elevated serum neuron-specific enolase (NSE) of >300 ng/mL (reference range: 1–16.3 ng/mL) and a lactate dehydrogenase (LDH) level of 1098 U/L. VMA and HVA data were not available for this retrospective review. Baseline blood counts and other biochemistry profiles were otherwise within normal limits. Baseline 18F-FDG PET/CT revealed a large retroperitoneal mass with metastatic involvement of the liver, lymph nodes, and skeleton (**Figure 1**). ^123^I-MIBG scintigraphy demonstrated diffuse uptake with a Curie score of 21. Bone marrow examination confirmed metastatic involvement. Molecular profiling revealed non-amplified MYCN, with no detec **Table 2** p36 or 11q deletions. Based on the INRG classification, she was diagnosed with stage M high-risk neuroblastoma.

**Table 2:** Summary table of clinical characteristics.

Variable	Patient 1	Patient 2
Age	4 years	3 years
Sex	Female	Female
MYCN status	Non-amplified	Non-amplified
Stage	M	M
Metastasis	Bone + marrow	Bone + marrow
Treatment	chemotherapy + naxitamab	chemotherapy + naxitamab
Response	CR	CR

The patient received the identical induction regimen as detailed for patient 1. Premedication with gabapentin was initiated 5 days prior to each naxitamab cycle, and granulocyte-macrophage colony-stimulating factor (G-CSF) was administered on days 6–10 of each cycle. During the first naxitamab course (Cycle 2 of induction), the patient developed grade 2 tachycardia, irritability, abdominal pain, and transient hypotension. These events resolved with supportive care and temporary infusion rate adjustments and recurred with similar severity during the subsequent 4 courses (Cycles 3–6). In addition, chemotherapy-related myelosuppression, manifesting as leukopenia, thrombocytopenia, and anemia, occurred during the post-chemotherapy recovery phase and was managed with rhGM-CSF and supportive transfusions as needed, with blood counts recovering prior to the next cycle. Detailed toxicity data are provided in **Table 1**.

Following six cycles of induction, her serum NSE normalized to 11.8 ng/mL (**Figure 2**). Post-induction ^123^I-MIBG scintigraphy demonstrated a Curie score decrease from 21 to 0, and 18F-mFBG PET/CT confirmed complete resolution of all prior lesions. Bone marrow minimal residual disease (MRD) assessed by multiparameter flow cytometry (sensitivity: 0.01%) was negative. Based on these concordant findings, the patient achieved complete remission per INRC criteria at the end of induction.

The induction phase concluded in 2025. She subsequently underwent single autologous hematopoietic stem cell transplantation in March 2026, followed by involved-field radiotherapy (total dose 21.6 Gy) to the primary site in May 2026. She is currently continuing consolidation therapy with naxitamab maintenance. At her most recent follow-up (6 months post-induction), ^123^I-MIBG scintigraphy revealed a Curie score of 0, with no evidence of relapse.

## Discussion

We report two children with newly diagnosed high-risk neuroblastoma (HR-NB) who received naxitamab concurrently with induction chemotherapy starting from the second cycle. Both patients successfully completed all six induction cycles and achieved complete remission per International Neuroblastoma Response Criteria at the end of induction, with Curie scores decreasing 0 in both cases. Published case reports documenting the feasibility and safety of naxitamab integrated into frontline induction chemotherapy remain scarce, as most published experience with chemoimmunotherapy during induction has involved dinutuximab (7, 8).This case report therefore provides practical clinical information on this specific regimen.

Notably, in both patients, the early introduction of naxitamab during induction did not interfere with peripheral blood stem cell collection, nor did it delay or preclude subsequent high-dose chemotherapy with autologous stem cell transplantation or involved-field radiotherapy. Both patients completed all planned consolidation procedures on schedule. This observation is clinically relevant, as any delay in stem cell collection or transplantation could jeopardize overall treatment outcomes. In terms of anti-tumor response, both patients achieved CR at the end of induction. Historically, in children with HR-NB treated with conventional induction chemotherapy without anti-GD2 immunotherapy, the CR rate at the end of induction has been reported to range from approximately 16% to 27% (9). While this comparison is indirect and our sample size is too small to establish efficacy, the concordant responses across imaging, MIBG, bone marrow MRD, and biochemical markers in both patients are encouraging.

The biological rationale for this approach is multifactorial. Unlike the post-consolidation setting, where immune function is severely compromised by high-dose chemotherapy and stem cell transplantation, the induction phase preserves innate immune effectors, particularly NK cells and macrophages, which mediate antibody-dependent cellular cytotoxicity, the primary mechanism of anti-GD2 antibodies (10). In addition, certain chemotherapeutic agents, such as cyclophosphamide and doxorubicin, may upregulate GD2 expression on tumor cells and promote immunogenic cell death, potentially enhancing antibody binding and anti-tumor immune priming (2, 11). Nevertheless, overlapping toxicities require careful attention, including chemotherapy-related myelosuppression that may compound cytopenias and naxitamab-induced capillary leak syndrome that demands vigilant fluid management. Our experience suggests that proactive supportive care, including GM-CSF priming and standardized premedication, can effectively mitigate these risks.

Our findings can be contextualized within the evolving landscape of chemoimmunotherapy for HR-NB. Furman et al. (12) reported encouraging outcomes with the humanized anti-GD2 antibody hu14.18K322A administered concurrently with induction chemotherapy in a phase II study, demonstrating a 3-year event-free survival of 73.7% and an end-of-induction response rate of 97%. Cupit-Link et al. (7) described a single-institution retrospective cohort of 27 newly diagnosed patients who received dinutuximab throughout all induction cycles, with an end-of-induction objective response rate (≥ partial response) of 96% and acceptable tolerability. More recently, the Children’s Oncology Group trial ANBL19P1 established the feasibility of delivering chemoimmunotherapy (irinotecan, temozolomide, dinutuximab, and GM-CSF) in the post-consolidation setting after tandem autologous stem cell transplantation (13). Collectively, these studies illustrate the expanding role of integrating anti-GD2 antibodies with chemotherapy across different phases of frontline therapy. However, none of these studies specifically evaluated naxitamab, a humanized anti-GD2 antibody with a substantially shorter infusion time (30–60 minutes) compared with dinutuximab, as part of the initial frontline induction regimen from cycle two onward in newly diagnosed patients without refractory disease. Thus, the novelty of our report lies not in the concept of chemoimmunotherapy per se, but in providing practical safety and feasibility data for naxitamab using this particular sequencing schedule in the upfront setting. These observations may offer a useful reference for clinicians considering naxitamab as an alternative to dinutuximab-based chemoimmunotherapy regimens, given its distinct infusion profile and toxicity management requirements.

The safety profile observed in our patients was consistent with the known toxicity spectrum of naxitamab (14–16). The most common adverse events were infusion-related pain, hypotension, rash, and capillary leak syndrome, all of which were grade 2–3 and manageable with supportive care and infusion rate adjustments. Notably, no patient required permanent discontinuation of naxitamab, and all symptoms resolved within 24–72 hours. We also observed that the frequency and severity of acute infusion-related reactions appeared to decrease over successive cycles, likely reflecting improved premedication, optimized supportive care, and growing familiarity with infusion management. A similar pattern was reported in the ANBL19P1 trial, where pain was most frequent during cycle 1 and generally improved over subsequent cycles (13). These experiences suggest that with appropriate premedication (including gabapentin and GM-CSF priming) and close monitoring, naxitamab can be safely integrated into multi-agent induction regimens.

We acknowledge several limitations. First, the two-case sample size precludes definitive efficacy conclusions. Second, the absence of a control group prevents attribution of the observed CRs specifically to naxitamab. Third, PET tracer heterogeneity at baseline and follow-up precludes quantitative cross-modality comparisons such as SUVmax. However, ^123^I-MIBG Curie scoring, the reference standard for bone assessment, was consistently performed, and the CR was supported by concordant findings across multiple domains, including imaging, MIBG, bone marrow MRD, and NSE. Fourth, the applicability to MYCN-amplified HR-NB remains uncertain. Fifth, the 6-month follow-up precludes assessment of long-term survival. In addition, urinary VMA and HVA levels were not available for the two patients in this retrospective review, as these biomarkers were not uniformly collected at diagnosis. Nevertheless, the diagnosis of HR-NB was unequivocally established by histopathology and MIBG scintigraphy in both cases, meeting the INRC diagnostic criteria.

Despite these limitations, our experience demonstrates that early introduction of naxitamab during induction chemotherapy is feasible and tolerable in children with HR-NB, without compromising subsequent consolidation therapy. This report provides practical safety data and clinical context for clinicians considering this sequencing strategy. Larger prospective studies are needed to define the optimal timing of anti-GD2 immunotherapy and to identify which patients are most likely to benefit.

## Conclusion

Early introduction of naxitamab during induction chemotherapy was feasible and well tolerated in two children with HR-NB, without compromising subsequent stem cell collection or autologous transplantation. Both patients achieved complete remission at the end of induction. Given the extremely limited sample size, these preliminary findings are hypothesis-generating rather than conclusive, but provide practical safety data to support further prospective evaluation of this sequencing strategy in larger cohorts.

## Data Availability

The original contributions presented in the study are included in the article/supplementary material. Further inquiries can be directed to the corresponding authors.
